# Analyzing Survival Rate of Leukemia Patients Applying Long Term Exponential Model

**DOI:** 10.31557/APJCP.2020.21.6.1539

**Published:** 2020-06

**Authors:** Mostafa Faridizadeh, Hamid Alavi Majd, Sayeh Parkhideh, Abbas Haji Fathali, Mehdi Raei, Nazanin Ramezani, Anahita Saeedi, Ahmad Reza Baghestani

**Affiliations:** 1 *Department of Biostatistics, Faculty of Paramedical Sciences, Shahid Beheshti University of Medical Sciences, Tehran, Iran. *; 2 *Taleghani Bone Marrow Transplantation Center, Shahid Beheshti University of Medical Science, Tehran, Iran. *; 3 *Hematopoietic Stem Cell Research Center, Shahid Beheshti University of Medical Sciences, Tehran, Iran. *; 4 *Health Research Center, Life Style Institute, Baqiyatallah University of Medical Sciences, Tehran, Iran. *; 5 *Department of Epidemiology and Biostatistics, Faculty of Health, Baqiyatallah University of Medical Sciences, Tehran, Iran. *; 6 *Physiotherapy Research Center, Department of Biostatistics, Faculty of Paramedical Sciences, Shahid Beheshti University of Medical Sciences, Tehran, Iran.*

**Keywords:** Leukemia, BMT, survival analysis, cure model

## Abstract

**Background:**

Making progressin treatment of all branches of cancers has increasedthe percent of patients that never experience the event of interest. These cases are called immune or cure and models for handling the data included cure fraction rate, are referred to as cure model or long-term survival models.

**Methods:**

The data for this historical cohort study, were collected from leukemia patients diagnosed between 2007 to 2014 and followed up until 2016 in Taleghani hospital and received BMT (Bone Marrow Transplant). Some data had to be excluded because of incomplete information. Using recorded files mostly and phone calls rarely, were made to confirm whether the patients were still alive or not. Death due to leukemia was regarded as interested event. Analysis were performed by R version 3.4.1and Stata version 14.

**Results:**

Number of recurrents after receiving BMT, pre-transplant Hb and age at diagnosis were found as significant prognostics of survival time. HD patients had the highest 5-years overall survival in category of diagnosis type with 81.3%. Cure fraction was estimated to be 64.1%.

**Conclusion:**

According to high percentage of censoring, using long-term model had better fit.

## Introduction

A group of diseases which cause abnormal cells to divide very quickly, is called cancer. These unhealthy cells can damage nearby tissues and infect them. It consists of lots of branches, starting in special parts of body (Bahannan, 2017). It is predicted that the cancer would be a major reason of death in the upcoming years (Bray et al., 2012a). Today Cancer is known as one of the most important causes of mortality in the world, with approximately 14 million new cases in 2012. Expected number of new cases is to be raised by about 70% in the next two decades. Cancer is the second leading cause of death worldwide, and also was the reason for 8.8 million deaths in 2015. Globally, nearly 1 in 6 deaths is due to cancer (Siegel et al., 2018).

Leukemia is a type of fast growing cancer which begins in the bone marrow. Also leukemia is a cancer of white blood cells. Leukemia is held for 2.5% of all new cases a cancer in 2012 and is the eleventh cause of death between all cancers. Leukemia is regarded as a common cancer. The number of new cases of leukemia was 352 per 100,000 men and women in 2012 (Bray et al., 2012b). Approximately 1.5 percent of men and women will be diagnosed with leukemia at some point during their lifetime, based on 2012-2014 data (Pejin and Karaman, 2017). The cause for most cases of this diseases not known (Wiemels, 2012), but some environmental factors such as ionizing radiations, chemical materials such as benzene, chemotherapy, smoking, genetic disorders, family history, financial and social level have been suggested as possible risk factors (Koohi et al., 2015).

Leukemia accounts for approximately 8% of all cancer cases and in all age groups in Iran similar to entire world and causes a significant death toll and heavy expenses for diagnosis and treatment processes (Koohi et al., 2015). 

Survival studies are the ones that response variable is time until the event of interest happens (like recurrent of a disease). Censoring is the determinative of survival studies that are actually either lost during the follow-up or are the cases who do not experience the event of interest during the period of study.As a result of progress in treating cancer in all branches, for some outcomes, there may be some cases who never face the event of interest (C. Lambert, 2007). The cases that never experience the event are called immune, cure or out of risk (Louzada et al., 2012). In these situations utilizing standard survival analysis methods such as Cox’sproportional hazard model or log-rank test does not seem to be appropriate because they do not account for the possibility of cure fraction (Arano et al., 2010). For example, in analyzing the rejection of a BMT, many cases may never reject it; therefore, a cured fraction for this population would exist. In such situations standard survival models such asCox proportional hazard or log-rank test may not work adequately because of refusing to take cure cases into account which leads to baiased estimates (Arano et al., 2010). Cure rate models (also known as long-term survival models) are special type of survival models, have been deployed for such situations that cure fraction exists (Maller and Zhou, 1996). Long-term survival models included two approaches known as mixture cure model introduced by Boag in 1949 and non-mixture cure model introduced by Chen et al., (1999). Cure rate is an important parameter in these models and may cause more accurate results for estimating survival time (Andersson et al., 2011). Exponential distribution(Kannan et al., 2010), Weibull distribution(Martinez et al., 2013), and so forth can be considered for survival time of long-term models.

The goal of this study was to investigate possible effects of risk factors on survival time of leukemia patients who received BMT.

## Materials and Methods

In the present study, patients diagnosed with leukemia, who received BMT, are analyzed. The dataset that has been collected in 9 months from patients all over Iranwith various types of leukemia, Non-Hodgkins Lymphoma (NHL), Hodgkin Disease (HD), Multiple Myeloma (MM), Acute Myeloid Leukemia (AML) andAcute Lymphoblastic Leukemia (ALL), candidates for receiving BMT, registered between 2007 to 2014 and followed up until 2016 in Department of BMT, Taleghani hospital affiliated to ShahidBeheshti University ofMedical sciences. Some of the records had to be eliminated because of incomplete information.Recorded files mostly and phone calls rarely, were made to confirmwhether the patients were still alive or not. Death due to leukemia was regarded as the event of interest. Time between diagnosis to death due to leukemia, considered as survival time. Risk factors were type of diagnosis, age, sex, BMI (Body Mass Index), level of pre-transplant hemoglobin (Hb) and number of recurrent after receiving BMT. Finally a total number of 427 patients were included in the study. To identify clinical characteristics of patients that might have had an impact on survival of the patients, a mixture cure rate modelwith variablesgender, age, BMI, number of recurrent after BMT pre-transplantHb, and type of diagnosis wasused, in addition to an exponential distribution for survival time and a geometric distribution for latent competing risk. This model is called Long-term Exponential Geometric (LEG) (Louzada et al., 2012). Analysis and figures were prepared using R version 3.4.1 and Stata version 14. Quantitative results were expressed as mean + standard deviation. The significance level was set at P ≤ 0.05.

## Results

We studied 427 patients including 234 men (54.8%) and 193 women (45.2%) who received BMT with various types of leukemia.The mean of survival time for the patients was 1548.9 days and 95% CI (1416.8, 1681.1). The 1-year, 3-years and 5-years survival rate, were estimated 96.1%, 81% and 73%, respectively. Number of cases who experienced the event (death) was 88 cases (20.6%).

**Figure 1:**
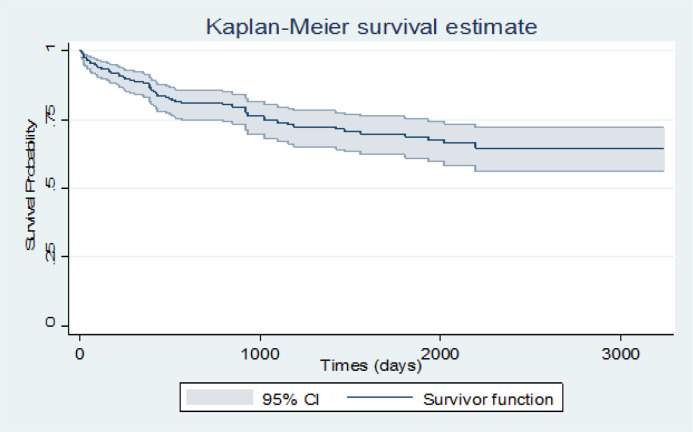
Survival Probability of Leukemia Patients for Total Study

**Figure 2:**
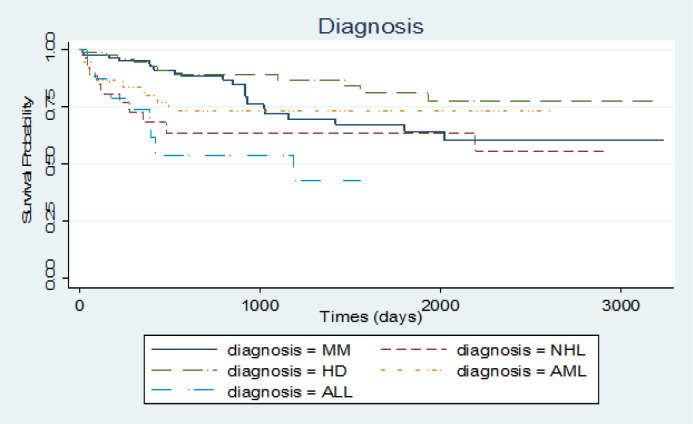
Survival Probability of Leukemia Patients for Category of Diagnosis Type

**Figure 3:**
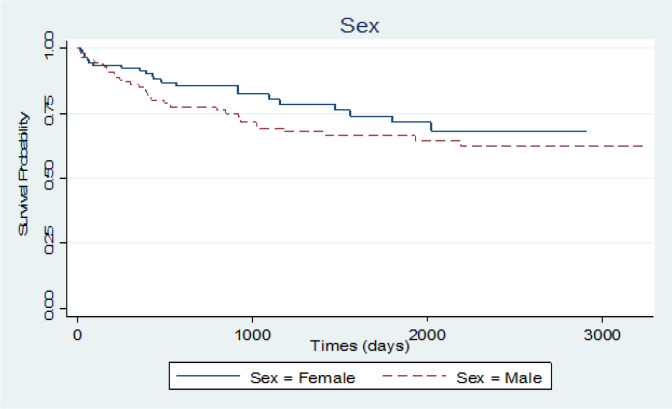
Survival Probability of Leukemia Patients for Category of Sex

To assess if data follows a cure rate pattern, Kaplan-Meier plot with a 95% confidence interval was conducted for this purpose. The plot reached a plateau after a while (about 6 years) and a 64.1% cure fraction was observed. Also Kaplan-Meier plot for survival probability of diagnosis type was applied and HD and ALL reached the highest and lowest survival time in this category with 81.3% and 43.1%, respectively. The same plots were executed for sex and age. In the sex category, women had a higher survival probability than men. Age variable was classified into two parts, less than 35 years and higher than 35 years and the group of younger than 35 years had a higher survival time (Figures 1, 2, 3 and 4).

The patients’ characteristics are listed in Table 1. The mean of age, number of recurrents after receiving BMT and pre-transplant Hb are 38.7, 0.2 and 10.9, respectively. Type of diagnosis, BMI and sex are categorical variables with 5, 4 and 2 categories, respectively. 

Estimated odds ratio of groups of MM, NHL, HD and AML diagnosis type to ALL group are 2.51(95% CI; 1.22-5.18), 1.68(95% CI; 0.85, 3.34), 4.62(95% CI; 1.63-13.1) and 3.35 (95% CI; 1.47-7.64) respectively.

Patients in MM, AML and HD groups have 2.51, 3.35 and 4.62 times more odds of being cured than patients in group of ALL, respectively, adjusting for other variables.

Estimated odds ratio of age was 0.91 (95% CI; 0.85, 0.99), it means for an increase of one year in age, the odds of being cured will decrease 9%, adjusting for other variables. 

Estimated odds ratio of number of recurrents after receiving BMT was 0.51(95% CI; 0.31, 0.83), again, it means for increasing one unit in recurrents after receiving BMT, the odds of being cured will decrease by 49%, adjusting for other variables. According to results of analysis shown in Table 2, age, number of recurrents after receiving BMT, pre-transplant Hb and diagnosis type found to be significant factors of survival time.

On the other hand the same significant difference observed for HD patients to NHL patients, HD patients to MM patients and AML patients to NHL patients. The other mutual diagnosis types showed no significant difference for odds of being cured (calculated but not shown).

**Table 1:** Patient’ Characteristics

Factor	Frequency	Percentage (%)
Diagnosis		
M.M	147	34.4
N.H.L	50	11.7
H.D	149	34.9
AML	49	11.5
ALL	32	7.5
Sex		
Male	234	54.8
Female	193	45.2
BMI		
UW (Under Weight)	24	5.6
NW (Normal Weight)	173	40.5
OW (Over Weight)	151	35.4
Obese	79	18.5

**Table 2:** Output

Factor	Estimate	SE	*P*-value	OR
Diagnosis				
MM	0.92	0.37	0.01*	2.05
A M L	1.21	0.42	0.004*	3.35
HD	1.53	0.53	0.004*	4.62
N H L	0.52	0.35	0.14	1.68
A L L				
Sex				
female	-------	-----	-------	
Male	-0.32	0.44	0.47	0.73
BMI				
UW (Under Weight)	0.37	0.69	0.59	1.45
NW (Normal Weight)	0.23	0.68	0.73	1.26
OW (Over Weight)	-0.61	0.81	0.45	0.54
Obese				
Age	-0.09	0.04	0.02*	0.91
Pre-transplant Hb	0.47	0.23	0.04*	1.6
Recurrent after	-0.68	0.25	0.006*	0.51

## Discussion

As a useful way to evaluate progresses in any type of cancer therapy, monitoring the trends in survival of patients over the time is suggested (Abu Bakar et al., 2008). In the present study time from diagnosis until meeting the event of interest has been measured. A long-term model using exponential function for survival time and a geometric function for latent variables was applied. 

This study assessed relationship between survival times of leukemia patients, who received BMT and some clinical, pathological, and biological variables. Based on our finding age, number of recurrents after receiving BMT and diagnosis types of MM and HD are significant factors.

Our study found age to be significant that is in favor of some other studies(Gupta et al., 2010; Popplewell and Forman, 2002).

Also results of present study showed a significant effect of number of recurrents after receiving BMT on survival time which is consistent with some earlier studies (Reshef et al., 2014; Wingard et al., 2011).

The effect of sex is not significantly approved by the result of our study that lines up with some studies (Gonçalves et al., 2009) and is not in range with some others (Kim et al., 2016).

Level of pre-transplant Hb had significant effect on survival time based on our data analysis which is supported by few existing studies (Xenocostas et al., 2003; Ditschkowski et al., 2004).

In this study there was no significant difference between survival time of BMI categories whichis lineto some previous studies(Aplenc et al., 2014), (Navarro et al., 2006).

Our work estimated 5-year overall survival of 58.4% for MM patients which is almost the same as previous studies. One of them reported a 5-year overall survival between33.8% to44.6% (Cornell and Kassim, 2016)and another reported a 6-yearoverall survival to be 45% (Kumar et al., 2014).

According to our study, 5-year overall survival of AML patients was 69.3%, which aligns with some other studies, 5-year overall survival of 71.9% (Shokouhi et al., 2015) and almost near 6-year overall survival were between 31% to 56% (Kumar et al., 2014).

In the present study 5-year overall survival of HD patients was estimated as 81.3% which is very close to 75% overall survival of HD patients after second remission (Lazarus et al., 2001).

Also, the 5-years overall survival of ALL patients reached 43.1% which is nearly the same as 5-years overall survival of 38% (Bishop et al., 2007) and 39% as 5-yearleukemia-free survival (Barrett et al., 1989) in former studies.

Estimation of the 5-year overall survival time of NHL patients was 56.8% which is similar to 71% (Schimmer et al., 2000) and 62% (Schimmer et al., 2000).

In the present study we used diagnosis type as a prognostic factor. Researches about leukemia has not been performed in a remarkable number (unlike breast cancer) and none of them mentioned evaluated types of diagnosis, so for assessing the effect of diagnosis types, overall survival time as a guideline was applied.

There were some limitations during accomplishment of the study. First of all, data was not prepared as ordered documents or recorded computer files to easily be gathered, so it has to be extracted from disorder documented files and also was done by statistical group (not medical group). In the other hand the data had not been integrated in a unique way, so gathering the data took a lot of time (about 9 months). Missing record and registering in wrong way caused a lot of excluding, hence a situation of a heavy deduction in data happened. 

Another problem was the deficiency of number of BMT surgeries. Numerous cases may improve the results.

Also changes in phone numbers (especially total changes of provinces) eventuated to zero communication with some cases and elimination of their information. 

In addition, we had no information about some other prognosis factors such as the socioeconomic, and family history of patients but interest may be done by investigating the impact of these factors.

Also the data was reached only by one center so the results extracted from this dataset could not be represent of all leukemia patients received BMT.

As previously mentioned about 70% of cases were cured, hence for leukemia patients, who received BMT,and did not experience the event, long term model could be considered as a good election. 

Further studies specially the ones covering limitations of this paper is advised.
